# Safety, Immunogenicity, and Efficacy of COVID-19 Vaccines in Radiation–Oncology Patients: A Systematic Review and Meta-Analysis

**DOI:** 10.3390/vaccines13070715

**Published:** 2025-06-30

**Authors:** Paul Thöne, Margot Egger, Michael Stephan Gruber, Georg Gruber, Christina Kasassov, Dalma Nyiri, Eva Weis, Helene Werl, Leonhard Trinkl, Wolfgang Lilleby, Martin Clodi, Elisabeth Bräutigam, Benjamin Dieplinger, Annette Aigner, Hans Geinitz

**Affiliations:** 1 Department of Radiation Oncology, Ordensklinikum Linz Barmherzige Schwestern, 4010 Linz, Austria; 2 Department of Medicine III, Medical University of Vienna, 1090 Vienna, Austria; 3 Department of Laboratory Medicine, Konventhospital Barmherzige Brüder Linz and Ordensklinikum Linz, 4010 Linz, Austria; 4 Medical Faculty, Johannes Kepler University, 4020 Linz, Austria; 5 Department of Orthopedic Surgery, Ordensklinikum Linz Barmherzige Schwestern, Vinzenzgruppe Center of Orthopedic Excellence, Teaching Hospital, Paracelsus Medical University Salzburg, 4010 Linz, Austria; 6 Department of Oncology, Oslo University Hospital, 0450 Oslo, Norway; 7 Department of Medicine, Konventhospital Barmherzige Brüder Linz, 4020 Linz, Austria; 8 CICMR-Clinical Institute for Cardiovascular and Metabolic Research, Johannes Kepler University, 4020 Linz, Austria; 9 Regional Health Agency of the State Lower Austria, Stattersdorfer Hauptstraße 6/C, 3100 St. Pölten, Austria; 10 Institute of Biometry and Clinical Epidemiology, Charité-Universitätsmedizin Berlin, Corporate Member of Freie Universität Berlin and Humboldt-Universität zu Berlin, Charitéplatz 1, 10117 Berlin, Germany

**Keywords:** SARS-CoV-2, cancer, vaccination, immunization, humoral response, cellular response, booster vaccination

## Abstract

**Background/Objectives**: The COVID-19 pandemic significantly threatened cancer patients and oncologic care. The rollout of vaccines emerged as a critical milestone, despite the initial lack of evidence regarding their safety and efficacy in this population. This systematic review and meta-analysis evaluate the current evidence on COVID-19 vaccination in patients undergoing radiotherapy (RT). **Methods**: PubMed, Livivo, Scopus, and Cochrane Library were systematically reviewed for relevant publications on COVID-19 vaccination in the context of radiation oncology, published by 19 April 2024. The treatment effects were calculated as the proportion of seroconverted individuals. **Results**: A total of 22 studies published between 2021 and 2024 were included, covering various aspects of vaccination, including safety, tolerability, qualitative and quantitative humoral responses, cellular responses, vaccination efficacy, and booster vaccinations. Notably, patients undergoing RT exhibited a high willingness to receive vaccination. Vaccination was overall well tolerated and safe, with a low incidence of side effects, which were primarily mild. The primary meta-analysis showed a seroconversion proportion of 91% [95% CI: 84–96%] overall, with a somewhat higher proportion of 93% in patients receiving RT alone, compared to 90% in patients receiving either RT or RT combined with chemotherapy. Furthermore, immunization during RT led to a sustained increase in antibody titers, with a notable long-term persistence of IgG. **Conclusions**: COVID-19 vaccines demonstrate excellent safety, immunogenicity, and efficacy in patients receiving RT, who also exhibit a high willingness to be vaccinated. The outcomes observed are comparable to those in healthy controls and superior to those seen in patients receiving other cancer treatments, such as chemotherapy. The vaccination of radiation oncology patients in future pandemics or epidemics is strongly advocated even during active treatment.

## 1. Introduction

The Coronavirus Disease 2019 (COVID-19) pandemic has profoundly affected radiation oncology as well as oncologic healthcare in general. Within the first year of the pandemic, reports indicated a notable decrease in both cancer treatments and new cancer diagnoses [1]. Concurrently, the number of radiotherapy (RT) sessions declined markedly [2]. Cancer patients were found to have an increased susceptibility to severe acute respiratory syndrome coronavirus 2 (SARS-CoV-2) infections [3] and were at higher risk of adverse outcomes [4].

The development of vaccines marked a crucial milestone in managing the pandemic and showed excellent efficacy and tolerability compared to placebos in healthy subjects [5]. In cancer patients, the use of COVID-19 vaccines was initially approached cautiously due to the lack of clinical evidence. However, subsequent research demonstrated their high efficacy and safety in the majority of patients with cancer [6,7]. Data on SARS-CoV-2 vaccination in patients undergoing RT were initially even more limited. An early review of the literature on RT and COVID-19 found that only 1.4% of the included publications addressed both RT and vaccination [8]. Since then, several institutions actively monitoring vaccine administration in the context of radiation oncology have published their results, contributing valuable evidence that could inform strategies for future pandemics.

This systematic review primarily focuses on current evidence regarding immunogenicity parameters in radiation oncology patients, specifically humoral immune response, cellular immune response, response persistency, and vaccination-related side effects. Additionally, a meta-analysis was conducted to pool findings on seroconversion (SC). A secondary aim was to systematically review the circumstances of vaccination, including willingness, hesitancy, and other factors influencing patient decision making.

## 2. Materials and Methods

**Figure 1:**
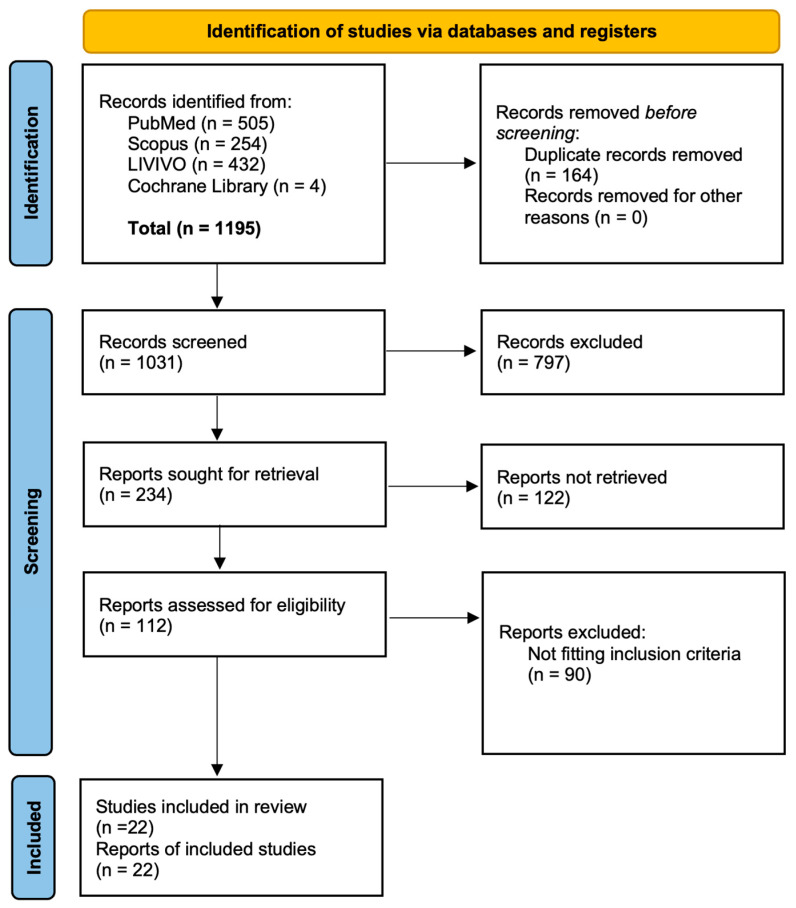
Flow chart of the study conception in detail.

**Study protocol.** A systematic review was conducted following the PRISMA (Preferred Reporting Items for Systematic Reviews and Meta-analyses) guidelines to minimize potential bias [9,10]. All included studies were evaluated using the Newcastle–Ottawa Risk of Bias assessment scale [11]. On 19 April 2024, the databases PubMed, LIVIVO, Scopus, and Cochrane Library were searched. The following search string was employed: >”radiotherapy” OR “radiation oncology” AND “SARS-coV-2” OR “COVID-19” AND “vaccin*” OR “immunisation” OR “immunization”<. The initial search yielded 1195 articles, from which 346 duplicates were removed, leaving 849 articles for screening. Following a multi-stage screening process—comprising title, abstract, and full text—22 studies were included (Figure 1). The inclusion criteria were as follows:

(1) The study investigated an oncologic population of individuals aged 18 years or older.
(2) Individuals received COVID-19 vaccination and underwent RT within the preceding 12 months.
(3) Vaccine administration preceded RT, except in studies considering the circumstances of vaccination, e.g., willingness, hesitancy, or decision-influencing factors.
(4) Immunological information, including side effects and/or data about the circumstances of vaccination, was reported.
(5) The study design corresponded to at least level II evidence, in line with the Oxford Centre for Evidence-Based Medicine [12].

Only original papers were considered, and literature reviews were excluded. The PICO search strategy was used to guide the literature review (Appendix A Table A1) [13]. The review process was conducted using CADIMA [14]. Of the 22 studies, 9 studies provided detailed SC data of COVID-19 vaccines in radiation oncology patients and were thus suitable for a meta-analysis. The study designs were classified as cross-sectional if patients were recruited after vaccination only, and as longitudinal if patients were recruited prior to vaccination and therefore recruitment and follow-up were at least two distinct time points.

**Statistical analysis.** Confidence intervals for proportions were calculated using the method of Clopper–Pearson [15]. The primary meta-analytic model for pooling SC proportions was a generalized linear mixed-effects model—additionally stratified by the additional use of chemotherapy as a cancer treatment, the time of RT, study type, and the exclusion of patients with previous SARS-CoV-2 infection. The inverse-variance method was used as a sensitivity analysis. Heterogeneity among studies was assessed using the following metrics: between-study variance Tau^2^, percent variation across studies due to heterogeneity I^2^, and a Chi-squared test of the null hypothesis of no between-study heterogeneity. The results from each meta-analysis are graphically presented using forest plots. Publication bias was assessed visually with a funnel plot. All statistical analyses were based on the software R (version 4.3.1) [16], just as in the R packages tidyverse and meta [17,18].

**Ethics.** No ethical approval had to be obtained for this study.

## 3. Results

For the systematic review of current evidence, relevant data were extracted from 22 studies [19,20,21,22,23,24,25,26,27,28,29,30,31,32,33,34,35,36,37,38,39,40] published between 2021 and 2024, all of which explored the intersection between vaccination and radiation oncology (Table 1). The majority of studies primarily analyzed general cancer patient samples, which included radiation oncology subgroups, while six studies exclusively investigated radiation oncology patients. Ten studies were conducted exclusively within cohorts of patients with solid tumors. Seven studies explicitly reported to have included a certain proportion of hematologic malignancies, varying from 4.3 to 33.0%. The studies evaluated various vaccines: BNT162b2 was administered in 15 studies, mRNA-1273 in 12, ChAdOx1 nCoV-19 in five, Ad26.COV2.S in six, and Sinovac in seven.

**Circumstances of vaccination.** Four studies reported data on vaccination circumstances [19,22,24,27,37]. Suzuki et al. observed that RT and/or chemotherapy patients adjusted their vaccination behavior according to their treatment schedules, opting to vaccinate or delay depending on planned therapies [37]. In a cross-sectional survey by Hong et al., among 82 patients receiving RT, 65 were vaccinated, while 17 declined [24]. Liu et al. similarly reported for a cross-sectional survey that more than half of RT patients (95 of 177) received vaccination. They presented only slightly lower odds for vaccination hesitancy compared to patients receiving other treatment modalities (unadjusted Odds Ratio (OR) = 0.932 [95% CI: 0.646–1.344], adjusted OR = 0.827 [95%CI: 0.510–1.340]) [27]. Geinitz et al. reported in their cross-sectional study a high willingness to vaccinate of 90.3% among RT candidates, with 15.5% receiving vaccination during antineoplastic therapy. Vaccination was declined by 9.7%, where the most common reasons for hesitancy included the intention to wait until treatment completion, indecision, distrust of the available vaccines, concerns about interactions with comorbidities, and prior infection [22].

**Safety and tolerability.** Six studies evaluated the tolerability of COVID-19 vaccines in RT patients [27,28,30,33,37,39]. Scoccianti et al. (2021) compared the side effects of mRNA-1273 in RT patients and healthy controls, finding similar patterns of early or late side effects attributable to the first or second dose [33]. Commonly reported side effects included fatigue, headache, pain at injection site, fever, chills, and redness at injection site, with higher incidence observed within 7 days after the second dose. However, RT patients experienced fewer side effects after both doses [33]. Thöne et al. reported excellent tolerability in patients during RT, with no severe side effects, and observed a positive association between the occurrence of side effects and humoral response kinetics [39]. Prayongrat et al. observed only mild side effects, particularly after the second dose of messenger ribonucleic acid (mRNA) vaccines, and a favorable tolerability in general [30]. Kian et al. found that three of nine RT patients experienced side effects, two of whom underwent RT alone and one combined with chemotherapy [28]. Liu et al. reported lower odds for side effects in RT patients compared to other treatment modalities (unadjusted OR = 0.478 [95% CI: 0.247–0.926]) [27]. Suzuki et al. highlighted patient anxiety related to the specific side effect of lymph node swelling, as they related it to potential cancer recurrence, especially in those with a history of lymphatic metastases. In this study, 37% of the 709 patients reported a swelling at the injection site [37]. Apart from original research articles, a unique RT-related side effect, the radiation recall phenomenon, has been described in some case reports as a consequence of COVID-19 vaccination [41,42,43,44].

**Figure 2:**
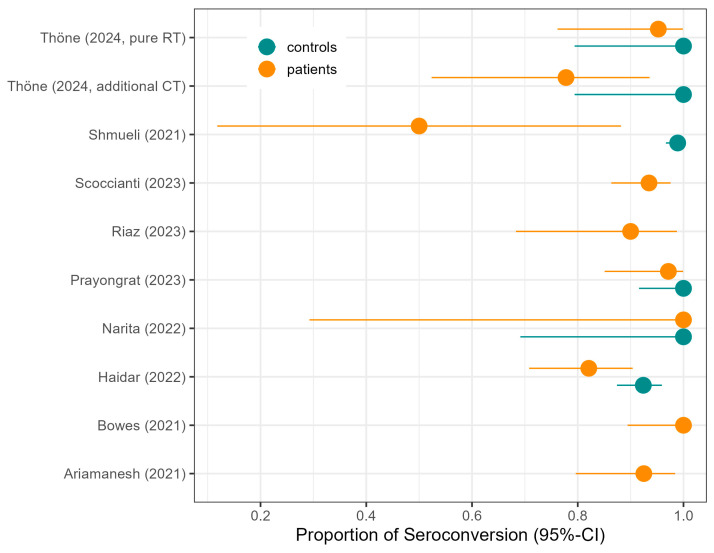
Proportion of seroconversion in patients as reported in the original studies of the meta-analysis, with seroconversion in controls where reported, along with 95% confidence intervals (CIs).

**Qualitative humoral vaccination response.** Ten studies assessed the humoral vaccination response of COVID-19 vaccines in cancer patients undergoing or having completed RT, out of which Joudi et al. [25] is, however, based on a subset of patients of Ariamanesh et al. [19] and therefore not included in the meta-analysis. Five of the nine studies included comparisons to healthy controls (Table 2, Figure 2).

Provencio et al. analyzed the association of seronegativity and cancer treatment modality. In detail, six months after vaccine administration, the adjusted odds for a negative serologic response were 1.46-fold [95% CI: 0.74–2.70] for RT patients compared to patients not receiving RT, and were also increased for patients receiving chemotherapy (odds ratio = 2.29 [95% CI: 1.12–4.85]) [31]. Scoccianti et al. (2023) observed that a younger age and breast irradiation were associated with a higher SC proportion [34]. Thöne et al. demonstrated a slower development of SARS-CoV-2-specific antibodies in RT patients, particularly in those receiving both RT and chemotherapy as compared to healthy controls [39]. Prayongrat et al. found an SC of 85% after a single adenoviral vaccination in RT patients, increasing to 89.5% after a second adenoviral vaccination, and 100% after a second immunization with an mRNA vaccine, while healthy controls achieved 100% [30].

Comparing pure RT to RT combined with chemoradiotherapy revealed higher SC proportions in RT-only patients. Ariamanesh et al. found that patients with RT only had higher frequencies of both binding (92.5% vs. 70.1%) and neutralizing antibodies (92.5% vs. 76.3%) compared with patients receiving chemotherapy with or without RT [19]. Joudi et al., which is a subset of the data in Ariamanesh et al., also described a higher SC in RT-only patients of 95%, compared to chemotherapy ± RT with an SC proportion of 66.7% [25]. These findings are in line with Thöne et al., who found that 95.3% of patients under pure RT seroconverted: 100% in healthy controls and 77.8% in radio-chemotherapy patients [39].

**Figure 3:**
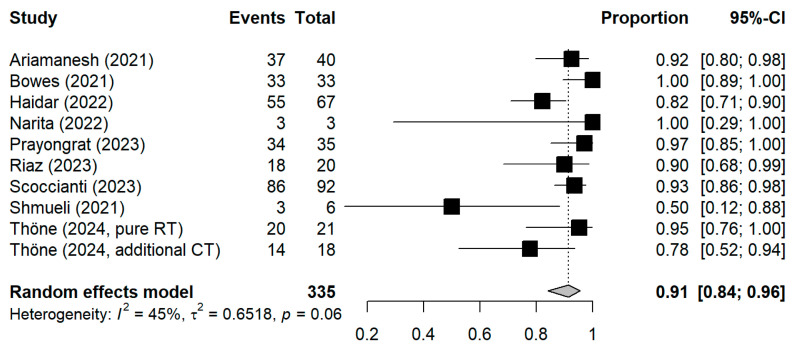
Forest plot of all studies included in the meta-analysis with the pooled result. Each square represents the point estimate with 95% CI for an individual study; The pooled estimate is shown as a diamond. CI = Confidence interval.

**Figure 4:**
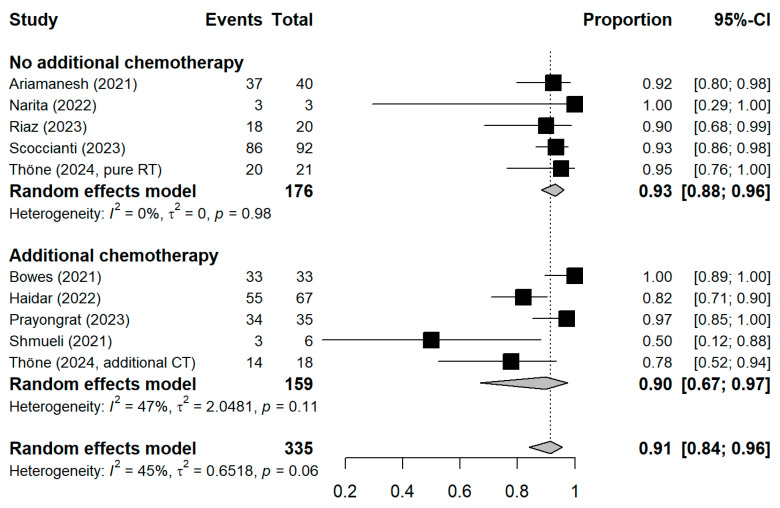
Stratified forest plot with the respective pooled result, discriminating between studies comprising patients receiving only radiotherapy and patients additionally receiving chemotherapy. CI = Confidence interval.

**Figure A1:**
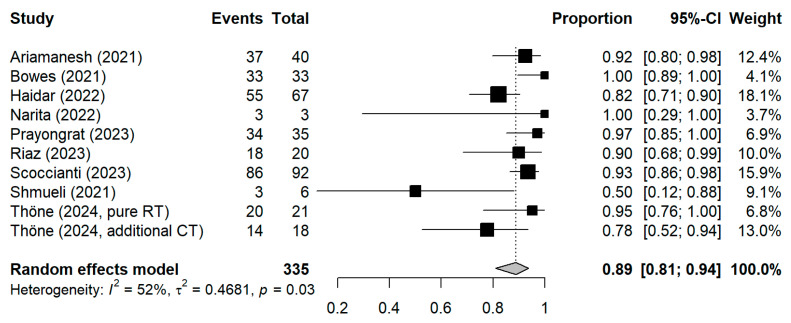
Forest plot of all studies included in the meta-analysis with the pooled result based on inverse-variance weights as a sensitivity analysis. CI = Confidence interval.

**Figure A2:**
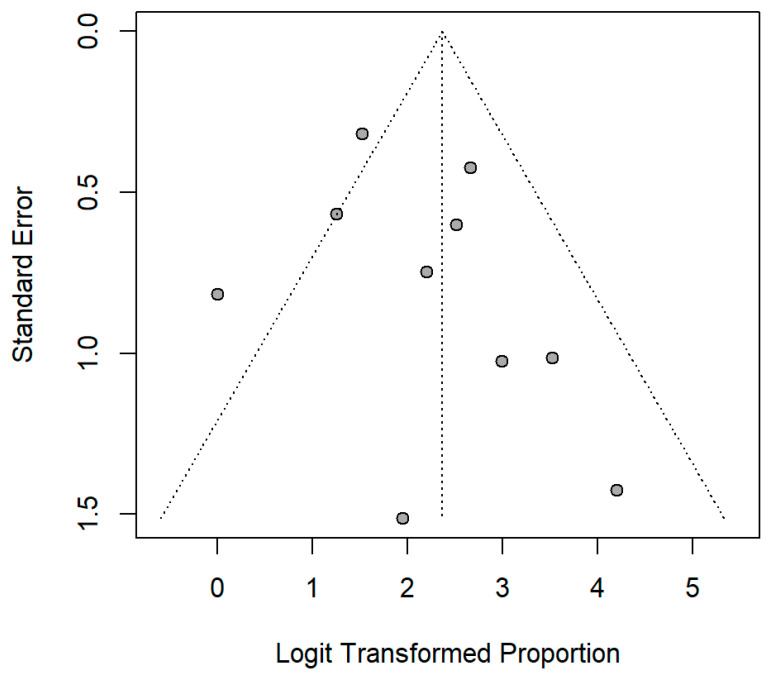
Funnel plot of the included studies.

**Figure A3:**
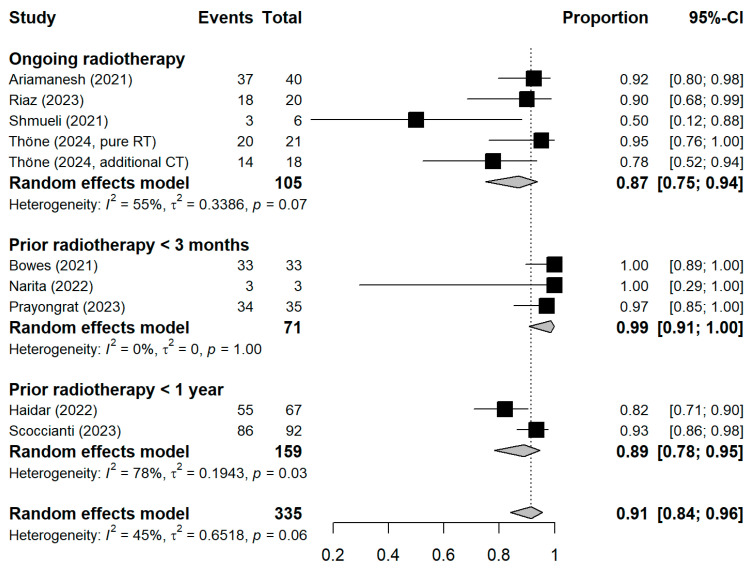
Stratified forest plot with the respective pooled result, discriminating between studies based on the temporal proximity of radiotherapy to vaccination. CI = Confidence interval.

**Figure A4:**
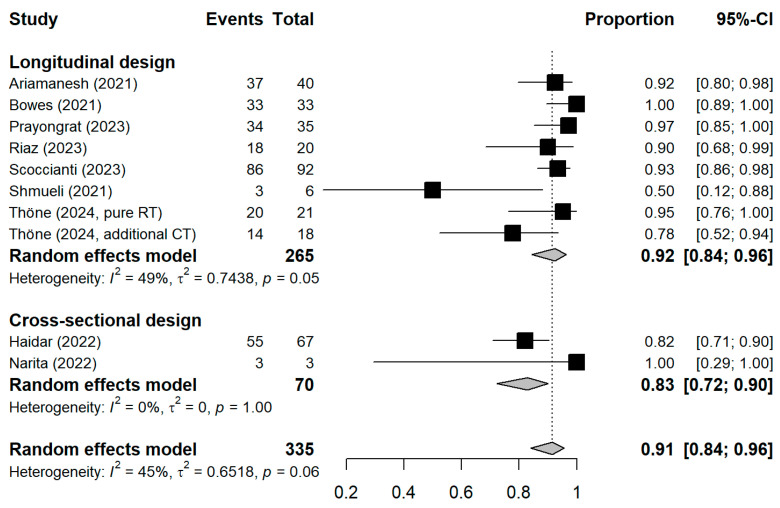
Stratified forest plots with the respective pooled result, discriminating studies based on the study design. CI = Confidence interval.

**Figure A5:**
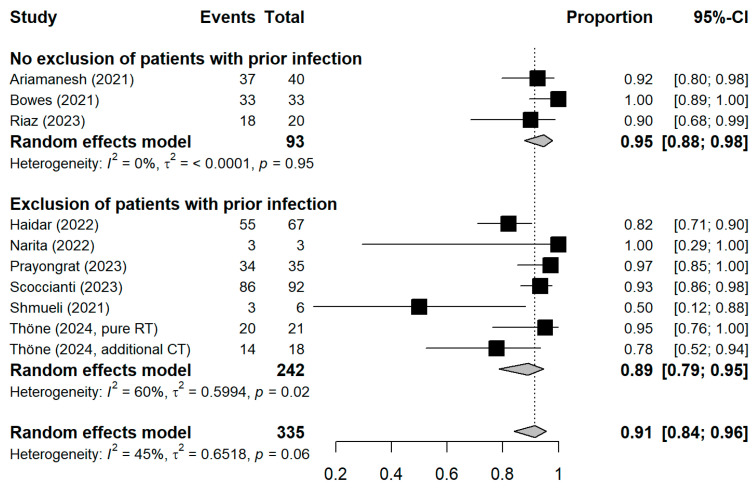
Stratified forest plot with the respective pooled result, discriminating between studies excluding or not excluding patients with a history of COVID-19 or positive nucleocapsid antibodies prior to vaccination. CI = Confidence interval.

The meta-analytic model based on all SC studies estimated a pooled SC proportion of 91% [95% CI: 84–96%] in RT patients, derived from a generalized linear mixed-effects model (Figure 3). Variability in the estimates was considerable (I^2^ = 45%, Chi^2^ test *p*-value = 0.06). Using inverse-variance weighting as a sensitivity analysis, the pooled SC was estimated to be 89% [95% CI: 81–94%] (Appendix A Figure A1). The funnel plot shows the potential of publication bias, as smaller studies with low proportions of SC may have not been reported (Appendix A Figure A2). Stratifying the meta-analysis by whether chemotherapy was given to the patients additionally to RT, we found somewhat higher SC proportions among RT-only patients with a much lower variability between the studies (I^2^ = 0%, Chi^2^ test *p*-value = 0.98). For those RT patients who additionally received chemotherapy, the proportion was somewhat lower at 90% [95%CI: 67–97%], with considerable heterogeneity (I^2^ = 47%, Chi^2^ test *p*-value = 0.11) (Figure 4). A further stratification indicated somewhat higher seroconversion proportions when the start of RT preceded vaccination by less than three months (ongoing RT: 87%, prior RT < 3 months prior: 99%, prior RT < 1 year: 89%) (Appendix A Figure A3). Stratifying by study design shows the tendency that cross-sectional studies report a lower seroconversion proportion (83%) compared to longitudinal studies (92%), however limited by the inclusion of only two cross-sectional studies (Appendix A Figure A4). A potentially more important design aspect is that most studies excluded patients with a history of COVID-19 or positive nucleocapsid antibodies prior to vaccination. As expected, these studies reported a lower seroconversion proportion (89%) than studies not mentioning this exclusion criteria (95%) (Appendix A Figure A5).

**Quantitative humoral vaccination response.** Six studies evaluated the quantitative humoral response [20,30,34,38,39,40]. Scoccianti et al. (2023) reported a median Immunoglobulin G (IgG) titer of 300 BAU/mL (range 7–1633) after a median of 147 days (range 144–154) after the second dose [34]. Uslu et al. found lower IgG levels in RT patients (mean: 1044.75 AU/mL, min–max: 2.00–40,000) compared to non-RT individuals (mean: 2278.00, min-max 353.20–40,000) [40]. Prayongrat et al. noted reduced anti-RBD total IgG levels in RT patients compared to those of healthy controls [30]. Thöne et al. observed similar mean titer values between pure RT patients and healthy controls at four (2001.34 IU/mL vs. 1640.06 IU/mL) and five (3165.71 IU/mL vs. 2994.13 IU/mL) weeks post-vaccination, although RT patients exhibited a slower initial increase [39]. Bowes et al. reported a geometric mean neutralizing antibody titer for RT patients 12 weeks after complete RT of 2.42 log10 U/mL [95% Cl: 2.13–2.72] for patients with thoracic malignancies who did not receive RT of 2.62 log10 U/mL [95% CI: 2.46–2.77], and 2.80 log10 U/mL [95% CI: 2.63–2.97] for healthy controls [20]. Antibody titers were inversely associated with immunosuppressive conditions, co-medication, chemotherapy, comorbidities, a palliative treatment intention [20], just as with age [34]. The concentration levels of anti-RBD total Ig were similar between patients receiving high-dose RT (>50 Gy) compared to low-dose (≤50 Gy) (e.g., three months after second dose, geometric mean of low dose: 115.4 [95% CI: 4.9–2734] vs. high dose: 142 [95% CI: 37.7–536.3]), according to Prayongrat et al. [30].

**Cellular response.** Two studies reported data on the cellular vaccination response [34,39]. In five sero-nonresponders, Scoccianti et al. (2023) found two positive and one borderline T-cell response out of five [34]. Thöne et al. assessed the T-cell immune response in four sero-nonresponders, all of which were negative [39].

**Vaccination efficacy.** In a registry-based case-control study, Lee et al. found that individuals with a recent cancer diagnosis, systematic cancer treatment, or RT within the past 12 months exhibited lower vaccine effectiveness against breakthrough and symptomatic SARS-CoV-2 infections [45]. In contrast, Seegers et al. reported significantly fewer breakthrough infections in cancer patients who underwent RT as compared to those who received other treatment modalities, such as chemotherapy [35]. Long-term measurements by Thöne et al., as well as measurements between the 2nd and 3rd dose by Scoccianti et al., found no nucleocapsid positivity, indicating an absence of viral exposure in these patients [34,39]. Bowes et al. concluded that RT does not interfere with the humoral vaccination response [20], while Prayongrat et al. considered effects of RT to be negligible [30].

**Booster vaccinations (third dose).** Six studies have reported findings of booster vaccinations [20,21,29,34,37,39]. Bowes et al. suggested the administration of an additional booster dose to vaccine non-responders in oncological patients [20]. In Thöne et al. booster vaccinations caused excellent results in terms of SC and good tolerability in all four individuals involved [39]. Similarly, Scoccianti et al. (2023) administered a third dose to 81 vaccinated individuals and observed a markedly enhanced humoral response of initially seronegatives, poor-, regular-, and ultra-responders [34]. However, Chen et al. investigated Sinovac booster vaccinations in lung cancer patients and found lower titers caused by booster vaccinations in RT patients than in other treatments [21].

## 4. Discussion

**Summary.** This review comprehensively evaluated the safety, immunogenicity, and efficacy of COVID-19 vaccination in oncology patients undergoing radiotherapy (RT). RT patients showed a high willingness to receive COVID-19 vaccinations, with acceptance rates between 79.3% and 90.3%, exceeding those of cancer patients undergoing other treatments. Vaccines were well tolerated, with only mild side effects. Quantitative humoral responses varied considerably across studies, with some patients showing reduced antibody titers, particularly those with immunosuppressive conditions or those undergoing chemotherapy, although the radiation dose itself had no impact. Findings on vaccine efficacy in RT patients were also heterogenous. While cancer patients, irrespective of treatment status, remain vulnerable to breakthrough infections, those undergoing RT demonstrated a significantly lower incidence of breakthrough infections compared to patients treated with chemotherapy or other treatments. Booster vaccinations are strongly recommended to optimize immunogenicity and reduce seronegativity, particularly in sero-nonresponders. The meta-analysis found a high pooled proportion of seroconversion of 91% in RT patients [95% CI: 84–96%], with higher and more consistent responses in those receiving RT alone. While early publications raised concerns that immunization during RT might impair immune response [7,46], later studies published during the pandemic supported vaccination for RT patients [8]. The results of this review and meta-analysis support the later studies in their conclusion.

**Comparison to Literature.** The high vaccination acceptance among RT patients found in this review, ranging from 79.3% to 90.3%, contrasts with a meta-analysis showing only a 59% acceptance among general cancer patients [47] and a Korean multicenter study reporting only 62% [48]. Identified major factors influencing hesitancy were treatment plans, timing, side effects, and uncertainties about efficacy and safety, which is consistent with prior findings from a systematic review and meta-analysis [47]. COVID-19 vaccines, however, demonstrated excellent tolerability in RT patients, with six studies reporting only mild to moderate side effects, comparable to the general population [49,50], and decreased reactogenicity has also been observed in cancer patients [51].

The meta-analysis found a high SC proportion of 91% among cancer patients who received RT, where patients receiving only RT had an even higher SC of 93%, compared to 90% with an additional treatment of chemotherapy. Other meta-analyses of cancer patient populations reported proportions of 73% [52], 90% in solid cancer and 63% hematologic cancer patients [26]. A self-conducted reanalysis of a meta-analysis by Yin et al. [53] estimated a pooled SC proportion of 49.5% (95% CI: 30.2–69.0%) for cancer patients after the first dose (vs. 90.6 (73.7; 97.1) in healthy controls) and 86.6% (95% CI: 81.8–90.3%) after the second (vs. 99.5% (98.4–99.9) in healthy controls). These findings suggest that although healthy controls have the highest proportions of SC, RT does not strongly compromise SC, especially not in comparison to other cancer treatments.

One study in this review showed that SC levels in RT patients remained stable over months [39], consistent with evidence that common Western vaccines (mRNA-1273, BNT162b2, Ad26.COV2.S, NVX-CoV2373) sustain humoral and cellular responses for over 6 months [54]. The findings on quantitative humoral response were heterogeneous, where some reported lower titers in RT patients [30,40], while others found not significantly different [38] or comparable titers to healthy controls [39]. In nine seronegative subjects pooled from two studies, a cumulative cellular nonresponse of 78% was found. Other meta-analyses of unselected cancer patients reported humoral response proportions of 78% at only 60% cellular response [52] or 79% humoral at only 61% cellular [55].

Reduced vaccine response in RT patients and the observed heterogeneity of findings highlight the importance of modifying factors such as prior or concurrent chemotherapy, immunosuppressive co-medications, poor overall health, and hematologic malignancies, as recognized both in the included studies [19,20,22,25,39] and previous meta-analyses [56,57,58]. Variability in SC and antibody titers may also be attributed to differences in vaccine platforms. For instance, systematically acting mRNA vaccines have demonstrated better performance compared to conventional vaccine platforms, both in RT patients [30] and in cancer patients more broadly [59]. Although RT is predominantly localized and induces primarily focal effects, systemic immunologic effects on lymphocyte subpopulations, blood cell count, and cytokine levels have been described [60,61].

Regarding booster or third vaccinations, an excellent tolerability as well as effectiveness/efficacy in overcoming seronegativity and increasing low antibody titers were observed. These findings are consistent with a meta-analysis of sero-nonresponding cancer patients, who achieved an SC proportion of 80% (solid cancer) and 44% (hematologic cancer) following booster doses [62]. Additional systematic reviews further supported the effectiveness of booster vaccinations in sero-nonconverting cancer patients, especially in those with solid tumors [63,64,65].

**Strengths and Limitations.** Following PRISMA guidelines, data from 22 studies were extracted and analyzed, and we used 9 to perform a meta-analysis on the proportion of seroconverted individuals, covering a broad range of vaccine platforms and cancer types. This review provides a comprehensive and systematic synthesis of the current evidence on COVID-19 vaccination in radiation oncology patients, encompassing vaccine safety, qualitative and quantitative humoral and cellular immune responses, and booster vaccination efficacy. The inclusion of real-world vaccination circumstances such as patient willingness and hesitancy strengthens the clinical relevance of the findings.

However, the interpretability of the findings is limited by the heterogeneity of the included studies, particularly regarding the intervals between RT and vaccination, as well as between vaccination and antibody measurements. Although these variations appear to have a minimal impact on SC, they may nevertheless corroborate the results. The potential for publication bias, suggested by asymmetry in the funnel plot, also limits the generalizability of the meta-analysis. Moreover, a separate analysis of vaccine platforms and types, or a consideration of patient characteristics such as co-morbidities and co-medications, was not possible.

## 5. Conclusions

This review demonstrated a high willingness among RT patients to receive COVID-19 vaccination and indicated that RT does not substantially impair vaccination response. Seroconversion was high, particularly in comparison to that in patients receiving other treatment modalities, and no evidence of increased adverse effects associated with vaccination in RT patients was observed. However, vaccine efficacy may be reduced in the presence of concomitant chemotherapy, immunosuppressive medication, or poor overall health status in cancer patients. As RT patients are particularly vulnerable to COVID-19 and require stringent protective measures, the vaccination of RT patients, even during active (pure) radiation treatment is strongly advocated, ideally under careful medical supervision. Especially patients receiving concomitant chemotherapy or those with other immunosuppressive conditions should undergo close monitoring of antibody levels following vaccination. Furthermore, the administration of booster doses in cancer patients receiving RT is strongly recommended, particularly in sero-nonresponders and in those with low antibody titers or absent cellular immune responses.

## Figures and Tables

**Table 1:** Studies included into the systematic review. The level of evidence according to the Oxford Centre for Evidence-Based Medicine classification.

No.	Study	Year	Country	N Total	N Pure Radiotherapy	Study Design	Level of Evidence	Source
1	Ariamanesh et al.	2021	Iran	364	49	Longitudinal	II	[18]
2	Bowes et al.	2021	USA	33	33	Longitudinal	II	[19]
3	Chen et al.	2023	China	260	17	Longitudinal	II	[20]
4	Geinitz et al.	2024	Austria	1142	1142	Cross-sectional	II	[21]
5	Haidar et al.	2022	USA	1099	61	Cross-sectional	II	[22]
6	Hong et al.	2021	China	2158	82	Cross-sectional	II	[23]
7	Joudi et al.	2022	Iran	160	20	Longitudinal	II	[24]
8	Lee et al.	2022	UK	2,258,553	not specified	Longitudinal	II	[25]
9	Liu et al.	2022	China	1132	117	Cross-sectional	II	[26]
10	Kian et al.	2022	Israel	210	9	Longitudinal	II	[27]
11	Narita et al.	2022	Japan	69	3	Cross-sectional	II	[28]
12	Prayongrat et al.	2023	Thailand	53	53	Longitudinal	II	[29]
13	Provenico et al.	2023	Spain	1973	186	Cross-sectional	II	[30]
14	Riaz et al.	2023	Pakistan	150	20	Longitudinal	II	[31]
15	Scoccianti et al.	2021	Italy	153	153	Longitudinal	II	[32]
16	Scoccianti et al.	2023	Italy	92	92	Longitudinal	II	[33]
17	Seegers et al.	2023	France	840	361	Longitudinal	II	[34]
18	Shmueli et al.	2021	Israel	129	6	Longitudinal	I	[35]
19	Suzuki et al.	2022	Japan	1182	20	Longitudinal	II	[36]
20	Takkar et al.	2021	USA	200	55	Cross-sectional	II	[37]
21	Thöne et al.	2024	Austria	46	46	Longitudinal	II	[38]
22	Uslu et al.	2023	Turkey	81	72	Cross-sectional	II	[39]

**Table 2:** Studies included in the meta-analysis of quantitative humoral vaccination response. The following vaccines were administered: ^1^ BNT162b2, ^2^ mRNA-1273, ^3^ ChAdOx1 nCov-19, ^4^ Ad26.COV2.S, ^5^ Sinovac.

Ref.	Study	Seroconversion (%)	N Total	N Pure Radiotherapy	Chemotherapy	Vaccine	Prior Radiotherapy	Cancer Types in Total Study Population	Serology After	Assay	Threshold
[18]	Ariamanesh 2021	92.5%	40	40	-	^5^	ongoing	solid 93.4%, hematologic 6.6%	2 months	ELIZA	8 mg/mL
[19]	Bowes 2021	100.0%	33	20	13	^1, 2, 4^	<3 months	solid 100%	≥2 weeks	Elecsys	0.8 U/mL
[22]	Haidar 2022	82.1%	67				<12 months	solid 91.0%, hematologic 9.0%	≥2_weeks	Beckman Coulter	0.8 U/mL
[28]	Narita 2022	100.0%	3	3	-	^1, 2^	<3 months	solid 100%	2–40 days	Lumipulse	
[29]	Prayongrat 2023	97.1%	35			^1, 2, 3^	<4 weeks	solid 100%	4 weeks	Elecsys	0.8 U/mL
[31]	Riaz 2023	90.0%	20	20	-	^5^	ongoing	solid 93.3%, hematologic 6.7%	2 months	ELIZA	8 µg/mL
[33]	Scoccianti 2023	93.5%	92	92	-	^1, 2^	<6 months	solid 88.1%, hematologic 4.3%, further	4–5 months	EliA	40 BAU/mL
[35]	Shmueli 2021	50.0%	6	5	1	^1^	ongoing	solid 100%	2–4 weeks	ELIZA	0.8 U/mL
[38]	Thöne 2024 Pure RT	95.2%	21	21	-	^1^	ongoing	solid 100%	2 weeks	ELIZA	0.8 U/mL
[38]	Thöne 2024 RT + Chemo	77.8%	18		18	^1^	ongoing	solid 100%	2 weeks	ELIZA	0.8 U/mL

## Abbreviations

The following abbreviations are used in this manuscript:

AU	Arbitrary Units
BAU	Binding Antibody Units
CI	Confidence Interval
COVID-19	Coronavirus Disease 2019
IgG	Immunoglobulin G
mRNA	Messenger Ribonucleic Acid
OR	Odds Ratio
RT	Radiotherapy
SARS-CoV-2	Severe Acute Respiratory Syndrome Coronavirus 2
SC	Seroconversion

## Appendix A

**Table A1:** Application of the PICO search strategy to guide the literature review.

PICO-Strategy.	
Patients	Cancer patients undergoing radiotherapy, aged 18 years or older
Intervention	COVID-19 vaccination administered before or concurrently to radiotherapy
Comparison	If applicable: healthy controls or other treatment modalities
Outcome	Immunogenicity and circumstances of vaccination
